# Bullseye dielectric cavities for photon collection from a surface-mounted quantum-light-emitter

**DOI:** 10.1038/s41598-023-32359-0

**Published:** 2023-03-31

**Authors:** Reza Hekmati, John P. Hadden, Annie Mathew, Samuel G. Bishop, Stephen A. Lynch, Anthony J. Bennett

**Affiliations:** 1 School of Physics and Astronomy, Cardiff University, Queen’s Buildings, Cardiff, CF24 3AA UK; 2 School of Engineering, Cardiff University, Queen’s Buildings, Cardiff, CF24 3AA UK

**Keywords:** Nanocavities, Single photons and quantum effects

## Abstract

Coupling light from a point source to a propagating mode is an important problem in nano-photonics and is essential for many applications in quantum optics. Circular “bullseye” cavities, consisting of concentric rings of alternating refractive index, are a promising technology that can achieve near-unity coupling into a first lens. Here we design a bullseye structure suitable for enhancing the emission from dye molecules, 2D materials and nano-diamonds positioned on the surface of these cavities. A periodic design of cavity, meeting the Bragg scattering condition, achieves a Purcell factor of 22.5 and collection efficiency of 80%. We also tackle the more challenging task of designing a cavity for coupling to a low numerical aperture fibre in the near field. Finally, using an iterative procedure, we study how the collection efficiency varies with apodised (non-periodic) rings.

## Introduction

The ability to engineer the emission characteristics of point-like emitters is crucial in the creation of single photon sources for applications in quantum computing and quantum communication^1,2^. The source’s photon collection efficiency (CE) and the emitter decay rate are two important figures of merit that can be tailored by cavity design. Numerous cavity designs have been thoroughly investigated such as photonic crystals^3,4^, micro-pillars^5,6^ and nano-antenna^7,8^.

Bullseye cavities, so called because of their resemblance to a bullseye target, consist of concentric rings of alternating dielectrics. These have recently attracted considerable attention due to their unique ability to enhance and direct emission from single photon sources into the far field over a broad spectral range^9–13^. These bullseye-cavities can offer enhancement to the photon collection efficiency and decay rate which is independent of the orientation within the plane of an ideally positioned emitter^14^. To date, reported bullseye structures are mainly dielectric-based and lossless, a key advantage over plasmonic cavities, which include absorptive metals in the high-field region^7,15,16^. These advantages have led bullseyes to be used to enhance the performance of quantum dots^8^, defect centres in diamond^17,18^, and in two dimensional (2D) materials^11,19^. Predicted values for spontaneous emission rate enhancement in the literature for the bullseye structures are as high as 56^20^.

Recently, a hybrid structure based on a bullseye has been reported in which a high Purcell factor over a broad range of wavelengths with high collection efficiency is achieved^21^. Inspired by the bullseye pattern, defects in photonic crystals were recently proposed as a new platform for extremely high Purcell factor with high collection efficiency^22^. A collection efficiency near unity can be achieved by utilising a low refractive-index SiO_2_ dielectric layer and a layer of gold as the mirror at the bottom of the structure^23^.

In this study, we demonstrate an approach to designing a bullseye structure to enhance the emission from point sources just above the centre of the structure. This case is applicable to emitters in WSe_2_ flakes laid over the cavity, single dye molecules or nano-diamonds applied to the structure. The resultant cavity achieves high collection efficiency (> 80%) and high Purcell factor (> 22.5) at the cavity resonance (750 nm). Subsequently, the coupling condition between the emitter and the cavity is discussed where this analysis underlines the importance of positioning in emitter-cavity systems. Finally, we study how the collection efficiency for coupling into a low numerical aperture fibre varies when the ring dimensions are apodised in an iterative manner.

### Cavity design

**Figure 1:**
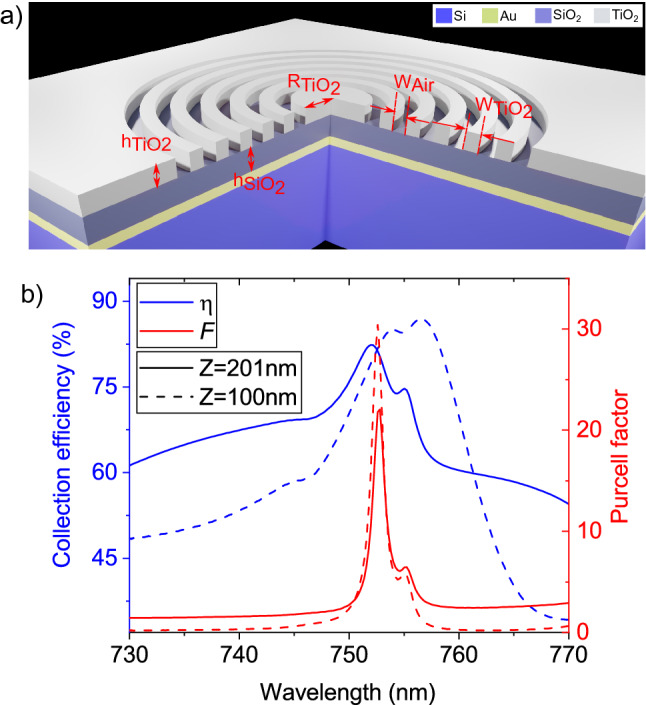
Bullseye dielectric cavity structure. (**a**) 3D representation of the structure, consisting of $TiO_{2}$ rings on a $SiO_{2}$ layer, above a gold mirror. The key dimensions of the structure are labelled. A dipole emitter is in the middle of the central disc. (**b**) Collection efficiency (left y-axis) and the Purcell factor (right y-axis) as a function of the wavelength for a 10 ring-pair cavity designed for 752 nm. Calculation performed with $R_{TiO_{2}} = 835\,\hbox {nm}, W_{Air} = 140\,\hbox {nm}, W_{TiO_{2}} = 280\,\hbox {nm}$. The height of the dipole above the surface of the silica is given by Z.

The structure in this work is depicted in Fig. 1a. It consists of a silicon substrate coated in a 150 nm reflective gold layer, $h_{SiO_{2}}= 435\,\hbox {nm}$ of SiO_2_ and $h_{TiO_{2}}$ = 200 nm of TiO_2_. A circular grating etched into the TiO_2_ layer consisting of a series of concentric rings centred around a disk with a radius of $R_{TiO_{2}}$. The width of each ring is $W_{TiO_{2}}$ and the distance between two consecutive rings is $W_{Air}$. The emitter is modelled as a dipole on the surface of TiO_2_ at the centre of a bullseye, at a height Z above the silica surface, using the Lumerical Finite Difference Time Domain (FDTD) package. The Au layer acts as a mirror that helps to redirect emission toward the collection optics.

A periodic circular grating, which satisfies the second-order Bragg condition for efficient vertical light extraction, should fulfil the phase matching condition for the periodicity, $\Lambda = W_{TiO_{2}} + W_{air}=m\lambda /2 n_{TE}$^14,24^. In this formula, *m*, $\lambda$ and $n_{TE}$ are an integer constant ($m=$1), the resonance wavelength and the effective transverse electric (TE) mode index propagating inside the slab, respectively. As explained in Ates et al.^24^ a fully etched grating provides better overlap of the guided field inside the slab and the etched region, higher guided wave reflectivity, and higher quality factor, leading to a higher Purcell factor. Therefore, $\Lambda =\lambda /n_{TE}$ is considered as the initial value for the grating period. Fig. 1b. shows the result of a simulation for a cavity with periodic rings as a function of the dipole wavelength, which achieves a maximum Purcell factor of 22.5 and a collection efficiency of 80% on resonance. In the following section we discuss in detail the design parameters explored to achieve the aforementioned cavity performance.

## Results

### Simulations of an ideal periodic circular Bragg grating

The circular Bragg grating exhibits high directionality of the radiated light from an emitter positioned in the centre of the structure. For concreteness, we refer to the collection efficiency, $\eta$, as the fraction of the total dipole power, normalised by the power emitted by the dipole, which is collected within a fixed numerical aperture (NA = 0.68), centred on the cavity.

In dielectric cavities the loss is considered to be negligible; therefore, the spontaneous emission rate enhancement is equal to the Purcell factor^25^. In experiments, the Purcell factor quantifies the reduction in the dipole transition’s radiative lifetime^26^. In simulations, the Purcell factor may be be determined by the ratio of the total radiated power of a dipole inside the cavity, divided by the total radiated power of the dipole in an uniform dielectric.

**Figure 2:**
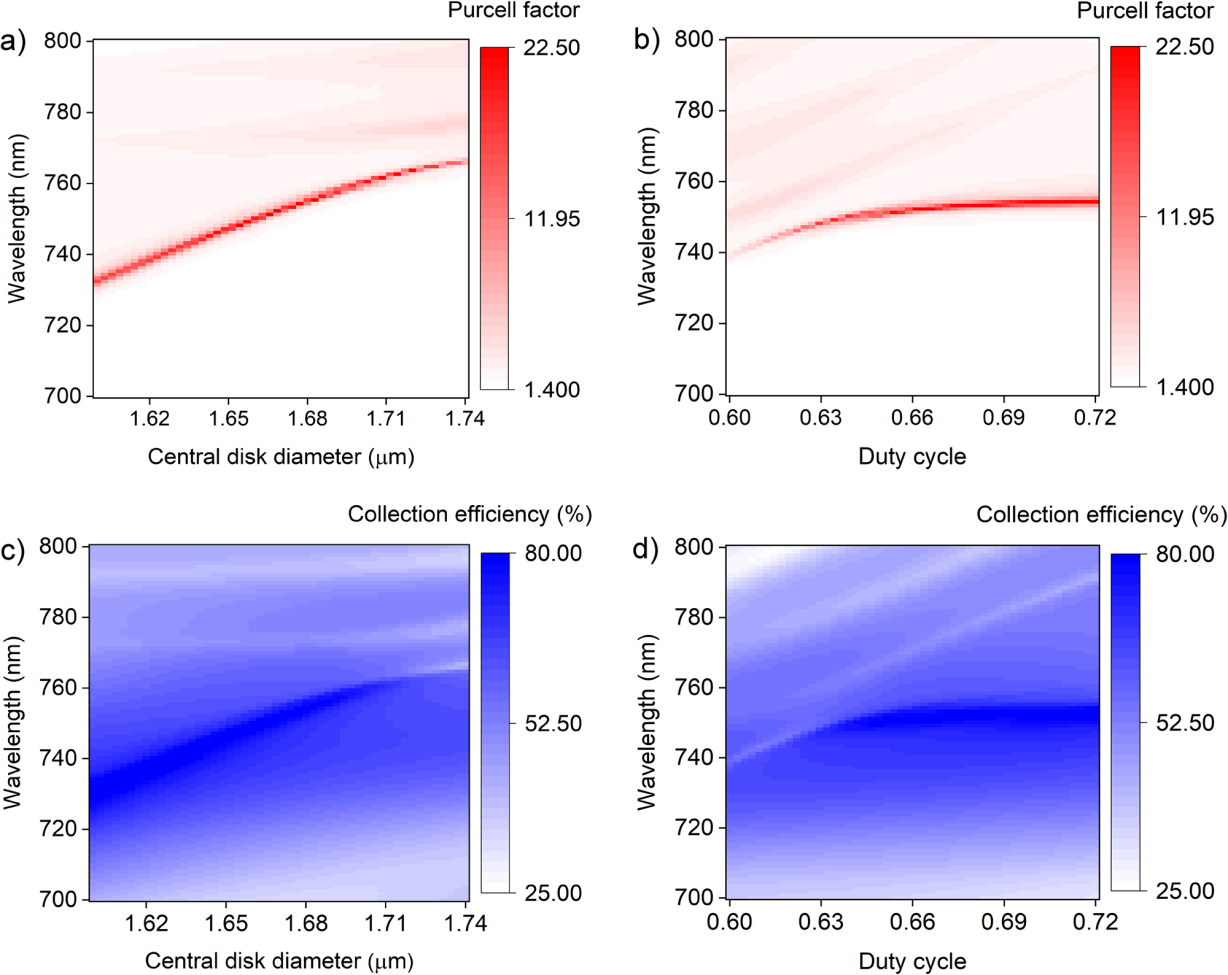
Bullseye cavity design. (**a**, **b**) Purcell factor as a function of the central disk diameter (**a**) and the duty cycle (**b**), and wavelength. (**c**, **d**) Collection efficiency into an ideal lens with an numerical aperture of 0.68 as a function of the central disk diameter (**c**), and the duty cycle (**d**) and wavelength. The optimal design for a resonance wavelength of 750 nm is achieved with a central disk size of 1.67 µm and a duty cycle of 0.66. The simulated cavity contains 10 rings-periods. (**a**) and (**c**) used duty cycle 0.67. (**b**) and (**d**) used $R_{TiO_{2}} = 1.67 \, \upmu \hbox {m}$.

Key parameters in the design of the structure are the grating period, $\Lambda =$ 420 nm, the duty cycle $W_{TiO_{2}}/\Lambda$ = 0.67, and the central disk diameter. Figure 2a–d illustrates the Purcell factor and collection efficiency, respectively, as a function of these parameters. We observe the resonances in the Purcell factor are narrower than in the collection efficiency; this means the Purcell factor is highly susceptible to the designed wavelength, highlighting the importance of accurate fabrication (also evident in Fig. 1b). In the case of the collection efficiency (Fig. 2c,d) the gold layer helps ensure some enhancement across a broad range of wavelengths. For a resonance wavelength of 750 nm the highest performance is achieved with $R_{TiO_{2}}=$ 835 nm and duty cycle 0.66. It is worth noting that the design parameters that correspond to the maximum Purcell effect do not correspond to the maximum collection efficiency, as visible in Fig. 2b and d below a duty cycle of 0.63. This highlights a key point in cavity design: enhanced scattering of light into a lens is a loss mechanism that can reduce the intensity of light trapped in the cavity, and therefore the Purcell effect.

### The dipole-cavity coupling condition

Considering that suitable design parameters have been determined, one can explore the coupling relationship between the dipole and cavity. The formula that explains this relationship is given by *F*, the Purcell factor^26–28^:

1 $$\begin{aligned} F = \frac{\tau _{free}}{\tau _{cavity}} = f(\Delta ). \left( \frac{\overrightarrow{d}.\overrightarrow{\varepsilon }}{|\overrightarrow{d}||\overrightarrow{\varepsilon }|}\right) ^2 . \frac{|\overrightarrow{\varepsilon } (\overrightarrow{r})|^2}{|\overrightarrow{\varepsilon }_{max}|^2}.F_{P} \end{aligned}$$

The first term in Eq. (1) describes the spectral detuning between the emitter and the cavity mode, and it is a function of the emitter-field spectral detuning ($\Delta$). The dipole-cavity relation in terms of the wavelength is depicted in Fig. 1b. When the dipole and the cavity are wavelength-matched, a Purcell factor of 22.5 and the collection efficiency of 80% are achieved. According to the Fig. 1b, detuning the dipole wavelength away from the cavity resonance presents, on average, a CE greater than 70% over a 4 nm bandwidth, and a CE above 60% over a 25.4 nm bandwidth.

**Figure 3:**
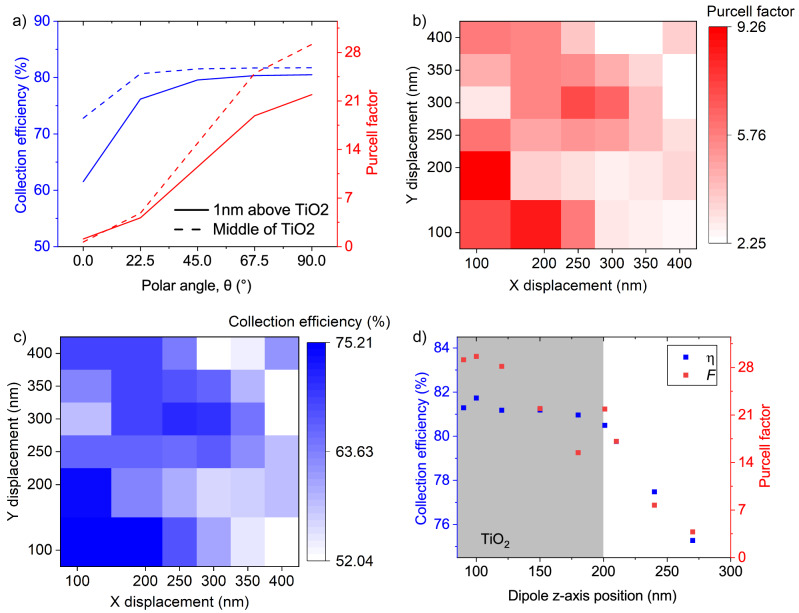
The effect of dipole orientation and displacement in the Bullseye cavity. (**a**) The Purcell factor as a function of the polar angle of the dipole. 0° and 90° represent out-of-plane and in-plane dipoles, respectively. (**b**) Purcell factor as a function of the dipole displacement in x and y-direction. (**c**) Collection efficiency as a function of the dipole displacement in x and y-direction. (**d**) Collection efficiency (left y-axis) and Purcell factor (right y-axis) as a function of the dipole position along the z-axis (x = y = 0). The system resonates at 752 nm. Number of rings in both simulations is 10.

The second term in Eq. (1) quantifies the alignment between the field strength and orientation ($\overrightarrow{\varepsilon }$) and the dipole strength and orientation ($\overrightarrow{d}$)^28^. The optimised structure was designed for an in-plane dipole, $\theta =90^{\circ }$, in which $\theta$ is the polar angle between the z-axis and the dipole. Collection efficiency and the Purcell factor are shown as red and blue in Fig. 3a. In the figure, two cases are investigated; when the dipole is placed 1 nm above the TiO_2_ layer (solid lines) and when the dipole is in the centre ($z=$ 100 nm) of the TiO_2_ disk (dashed lines). A 1 nm displacement above the device might be achieved by encapsulating the 2D emissive layer within hBN. We see that the higher the angle of $\theta$, the better the value of the collection efficiency and the Purcell factor. However, even for a dipole with vertical orientation the CE is above 60% which is 2 orders of magnitude greater than in the absence of the cavity. The emission pattern for a vertical dipole in free space follows a sin-squared dependence, preferentially emitting into the plane. This is what the bullseye is designed to deal with: the periodic nature of the rings scatters light out of plane to be collected by the lens. The fact that the collection efficiency is so high attests to the good performance of the resonator in combination with a high numerical aperture lens.

The third term in Eq. (1) describes the spatial detuning of the emitter with respect to the cavity as expressed by the ratio of the electric field ($\varepsilon$) at the emitter spatial position *r*, relative to the maximum electric field ($\varepsilon _{max}$). Figure 3b and c shows the Purcell factor and the collection efficiency as a function of the displacement in x and y directions, respectively. The Purcell factor drops by half when the dipole is displaced 70 nm from the centre. It should be noted that the x and y displacement plots are not the same because the dipole is oriented along the x axis. The collection efficiency (blue) and the relative Purcell factor (red) are also shown as a function of displacement in the z-direction, for x = y = 0, in Fig. 3d. As one might expect, the Purcell factor is greatest at the centre of the TiO_2_ disk ($z=$ 100 nm), but an appreciable enhancement can be seen when the emitter is at the surface. Finally, $F_{P}$ is a figure of merit representing the maximum enhancement of the spontaneous emission rate for a source with zero spectral detuning, optimal polarisation orientation and ideal positioning of the emitter relative to the mode. When all conditions are achieved the maximum coupling between the emitter and the cavity can be expressed by $F_{P}= 3Q(\lambda _{cav}/n)^3/4\pi ^2V_{eff}$, where *Q* and $V_{eff}$ are the quality factor and the effective mode volume of the cavity, respectively^29^. We defined the mode volume (*V*) as $\int \varepsilon E^{2} dV/max(\varepsilon E^{2})$ and according to our calculation, $V \sim 0.076 \, \upmu \hbox {m}^{3}$^30^.

### The effect of increased cavity periods

To quantify the effect of increased cavity periods, the effective enhancement factor (EF)^31^ (or the brightness enhancement^21^) is investigated. It includes not only the enhancement of the emission, but also the excitation. It is defined as:

2 $$\begin{aligned} \langle EF\rangle _{eff} \propto \frac{\gamma _{exc} (\lambda _{exc})}{\gamma ^{0}_{exc}(\lambda _{exc})} \times \frac{QE(\lambda _{em})}{QE^{0}(\lambda _{em})} \times \frac{\eta (\lambda _{em})}{\eta ^{0}(\lambda _{em})} \end{aligned}$$

In the above equation, the parameters with “0” in the superscript indicate the reference structure which is defined as the planar layer of TiO_2_ on SiO_2_/Au/Si substrate.

**Figure 4:**
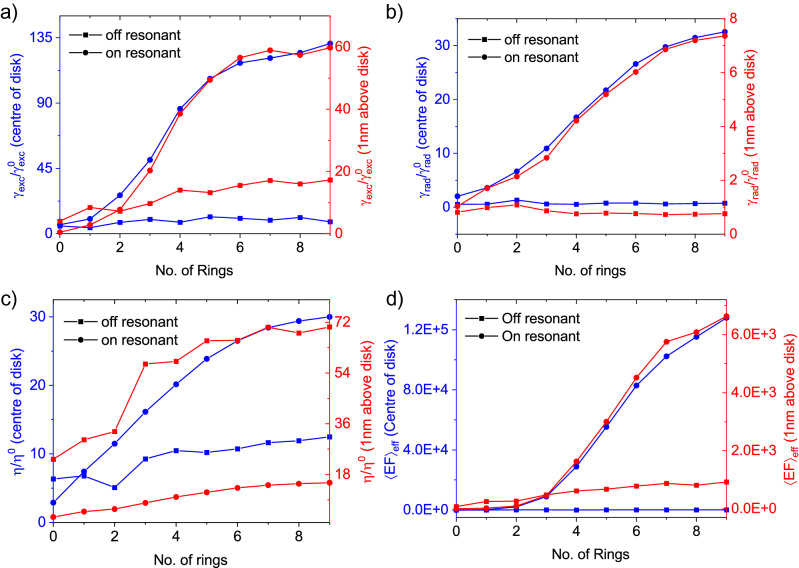
Dipole-cavity coupling efficiency. (**a**) Excitation enhancement rate for a dipole in the centre of the cavity (red) and a dipole 1 nm above the disk (blue) as a function of the number of rings. Square and circular shapes illustrate off-resonance (532 nm) and on-resonance (752.5 nm) excitation, respectively, exciting the structure with a plane wave and monitoring the field intensity. (**b**) The radiative decay rate enhancement as a function of the number of rings. (**c**) The collection efficiency enhancement as a function of the number of rings. In all plots, one can observe the general trend of increasing performance of the cavity with more rings. (**d**) Effective enhancement for a dipole positioned at the centre of disk (blue) and a dipole positioned 1 nm above the disk (red). In the simulations on a device with zero rings it still contains a central disc of dielectric with $R_{TiO_{2}} = 1.67 \, \upmu \hbox {m}$ which may enhance the figures of merit.

The first term in Eq. (2) is an enhancement ratio of the excitation for the bullseye structure compared to the reference structure when the structures are excited by a plane wave and the field is monitored at the point of the emitter. Figure 4a depicts this term in the above equation. Two excitation wavelengths were investigated; 532 nm (relevant to off-resonance excitation) and 752.5 nm (on-resonance excitation, at the wavelength of the cavity). At both wavelengths, increasing the number of rings leads to a greater enhancement factor; however, when the dipole is placed 1 nm on top of the disk, resonance excitation enhances by a factor of $\sim$ 3.45 in comparison to off-resonance excitation. For the dipole embedded at the centre of the disk, this ratio is approximately 15.9.

The second term in Eq. (2) quantifies the ratio of the quantum efficiency (yield) enhancement at the wavelength of the emission. Quantum efficiency in the emitter-cavity system is defined as $QE=\gamma _{rad}/(\gamma _{rad}+\gamma _{nrad}+\gamma _{loss})$ in which $\gamma _{rad}$, $\gamma _{nrad}$, and $\gamma _{loss}$ are radiative decay rate, non-radiative decay rate, and loss, respectively. In dielectric cavity systems, the loss is negligible. For emitters with a poor intrinsic quantum yield like emitters in exfoliated 2D materials^32^, it can be assumed that $\gamma _{nrad} \gg {\gamma ^{0}_{rad},\gamma _{rad}}$; therefore, the quantum efficiency is simplified to $QE/QE^{0}=\gamma _{rad}/\gamma ^{0}_{rad}$. Figure 4b shows the relative decay rate enhancement as a function of the number of rings.

The final term in Eq. (2) is the relative collection efficiency. The collection efficiency enhancement ($\eta /\eta ^{0}$) is shown in Fig. 4c. Increasing the number of rings increases these separate figures of merit. The product of these terms results in the enhancement of the photon rate extracted under CW excitation. For on-resonant (off-resonant) excitation, the enhancement factor for the dipole at the centre of the disk tends to a value of 14,500 ($\sim$ 910) as the number of rings increases whereas the same value for a dipole 1nm above the disk is around $\sim$ 6610 ($\sim$ 1910). (shown in Fig. 4d).

### Increasing collection efficiency into an optical fibre by apodization

The fact that bullseye cavities are flat, rotationally symmetric and highly directional makes them suitable for direct coupling to single mode fibre. Indeed, directly bonding fibres to these efficient structures could be a route to creating robust, compact and connectorised quantum light sources used in real world applications^33–35^. However, to obtain high collection efficiency the cavity must match the mode field diameter and NA of the fibre. Here we design a cavity for this application with concentric non-periodic rings, which allows us to vary the mode extent and NA. We refer to this variation of ring dimensions as apodization. The large number of variables in the design makes finding a global maximum efficiency non-trivial. Therefore, we approach this problem by iteratively varying each ring in turn, as discussed below. We consider a commercially available single-mode fibre, Thorlabs 630HP, with a core radius of 1750 nm and core (cladding) extending outside of the simulation region, refractive indices of 1.46 (1.45) and NA = 0.13, fixed above the structure. To calculate CE we summed far field emission within the numerical aperture of 0.13 and compared it to the emission into all directions.

**Figure 5:**
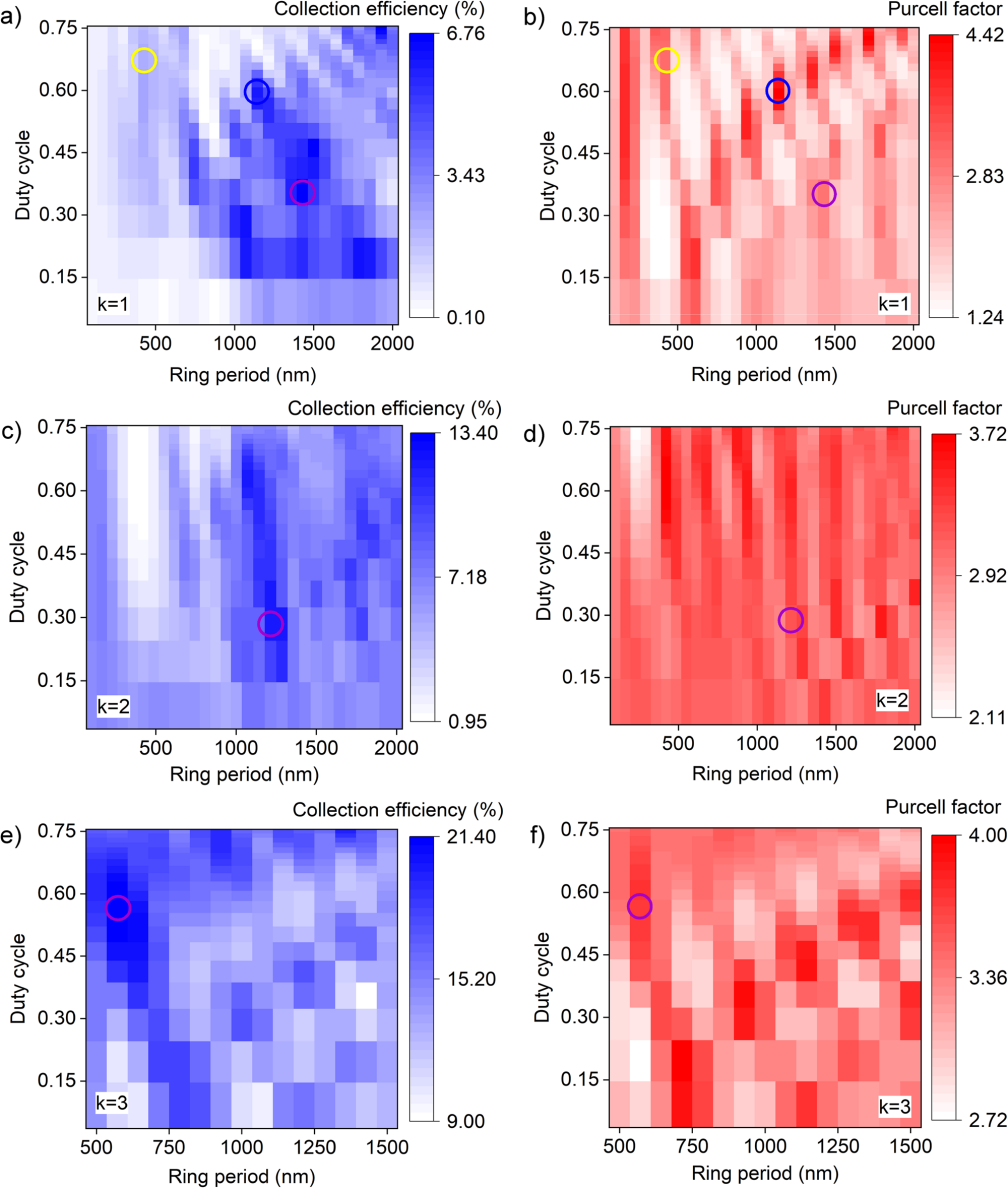
Apodization results for the bullseye structure. (**a**, **c**, **e**) Collection efficiency and (**b**, **d**, **f**) Purcell factor as a function of ring period and duty cycle for the first ring (k = 1), the second ring (k = 2), and the third ring (k = 3), respectively. Circles highlight points where the design meets the Bragg condition (yellow), the highest collection efficiency (purple) and the highest Purcell factor (blue). After the collection efficiency is maximised for a ring, it is fixed in dimension before next ring is added and optimised. For these simulations the fibre is included 2 microns above the bullseye and the field imaged 3 microns above the bullseye. This field is used to generate the far field emission pattern.

Figure 5a depicts collection efficiency as a function of ring period and duty cycle for a cavity with a fixed central disk dimension and only one ring (k = 1). Three areas are highlighted with circles; the yellow circle shows the result for a the single ring that meets the Bragg condition, giving collection efficiency of 2.2%. The purple circle indicates the highest collection efficiency of 6.7% (ring period 1428 nm, duty cycle 0.36). The blue circle indicates the highest achieved Purcell factor of 4.4 (ring period 1100 nm, duty cycle 0.60, Fig. 5b). Again, we notice that the highest collection efficiency does not coincide with the highest Purcell factor, as the additional loss implied by efficient photon collection leads to a lower field in the cavity. We aim to achieve higher collection efficiency, therefore we fix the ring period at 1428 nm and duty cycle 0.36 for the subsequent stages of the iterative design process.

**Figure 6:**
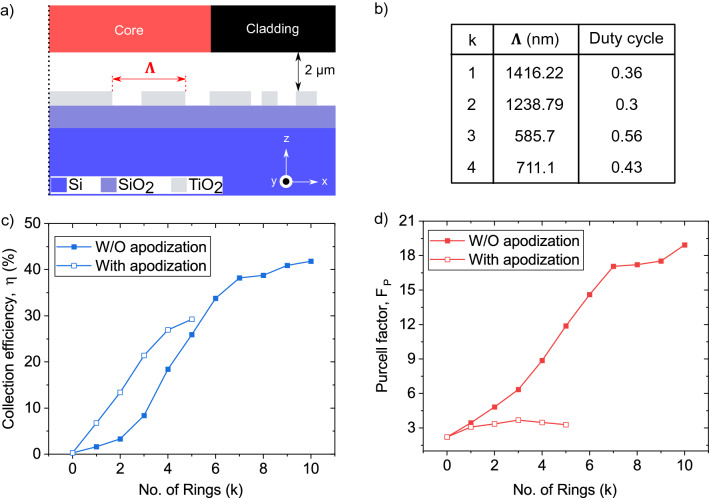
Apodised dielectric bullseye cavity coupled to single mode fibre. (**a**) A cross-section of the apodized bullseye structure. Vertical dotted line shows the symmetry axis of the rings. (**b**) Apodized values for the ring period ($\Lambda$) and duty cycle used in this work. (**c**) Collection efficiency and (**d**) Purcell factor as a function of the number of rings (k). In all simulations, the distance between the fibre and the bullseye structure was constant (2 μm).

We then include a second ring and vary its dimensions, as shown in Fig. 5c and d. The blue circle highlights the highest collection efficiency of 13.4% for the ring period and duty cycle of 1214 nm and 0.28, respectively. Fixing the dimensions of the second ring, one can then vary the dimensions of the third ring, as shown in Fig. 5e and f. We continue with this iterative process up to 5 rings, and the results are shown in Fig. 6c compared to the figures of merit for a Bragg cavity with the a same number of rings. Apodisation leads to higher collection efficiencies than that of the uniform Bragg cavity for 5 or fewer periods. However, the increasing collection efficiency as a function of the number of rings saturates around k = 5. This is to be expected, as at this point the extent of the rings is outside the mode field diameter of the fibre. Figure 6a and b summarises the result of this iterative optimisation on the period and duty cycle and Fig. 6d shows the resulting Purcell factor for the apodized and non-apodized structures. Underlining the trade-off between achieving high efficiency and high Purcell effect, we see that the efficiency-optimised apodized structure has a lower Purcell effect than the Bragg cavity. It may be possible to further increase the collection efficiency by introducing a non-uniform etch between rings^24^ or a mode matching element or dielectric between the cavity and the fibre^20^.

## Discussion

We have investigated the ability of “bullseye” cavities to enhance an emitter on the surface of the structure, which is relevant for future work in nano-optics and quantum technology with 2D materials, dye molecules and nano-diamonds. These are all physically interesting systems that may form key elements in future quantum technologies, especially if one can make efficient, fibre coupled quantum light sources. Future research could focus on the ability of these cavities to be robust to fabrication imperfections in the definition of the etched rings or their sidewall roughness. The design challenge of optimising the bullseye parameter with the multi-parameter optimisation (computationally intensive), or step-wise optimisation (prone to being trapped in local minima) may better solved by an approach based on inverse design, machine learning or a genetic algorithm, which would be an interesting topic of future work.

## Data Availability

Data supporting the findings of this study are available in the Cardiff University Research Portal at 10.17035/d.2022.0218102868.
